# Streptococcal Toxic Shock Syndrome Caused by Cellulitis Following a Fall: A Report of Two Critical Cases

**DOI:** 10.7759/cureus.80806

**Published:** 2025-03-19

**Authors:** Yoshitaka Saegusa, Satomi Yamamoto, Ayaka Shincho, Hitomi Furuoka, Jhun Hasada

**Affiliations:** 1 Infection Control Team, Merry Hospital, Hiroshima, JPN; 2 Department of Gastroenterological and Transplant Surgery, Graduate School of Biomedical and Health Sciences, Hiroshima University, Hiroshima, JPN

**Keywords:** cellulitis, elderly patients, japan stss outbreak 2024, necrotizing soft tissue infection (nsti), streptococcal toxic shock syndrome (stss), streptococcus pyogenes infection

## Abstract

Streptococcal toxic shock syndrome (STSS) is a life-threatening condition characterized by a sudden onset and rapid progression to multiple organ failure. The pathogen involved is Group A streptococcus (GAS).

We report two cases of STSS that developed following post-traumatic cellulitis in elderly individuals residing in an assisted living facility. The first case involved a 93-year-old male. He developed cellulitis in his right leg after a fall, which rapidly progressed to multiple organ failure. Despite antibiotic therapy, he succumbed to the infection within 30 hours of admission. Blood culture detected GAS. The second case was a 72-year-old man. He developed cellulitis after falling and hitting his leg. He initially received oral antibiotics for cellulitis, but his condition worsened rapidly, requiring emergency amputation and intensive care. Although he temporarily recovered, he later developed severe pneumonia and passed away. A culture of necrotic tissue obtained during surgery confirmed the presence of GAS.

STSS is an invasive infection with a high mortality rate, often triggered by minor skin injuries. Although STSS following minor trauma has not been previously reported, our findings suggest that even minor wounds from falls can serve as an entry point for GAS, leading to severe infection. Given the increasing prevalence of STSS in Japan, healthcare providers, particularly in facilities caring for elderly individuals, should maintain a high index of suspicion for early detection and intervention.

We present two cases of STSS following post-traumatic cellulitis. With the rising incidence of STSS in Japan, early recognition and prompt treatment are crucial in all healthcare settings to improve patient outcomes.

## Introduction

Streptococcal toxic shock syndrome (STSS) is a life-threatening disease characterized by sudden onset and rapid progression to multiorgan dysfunction, even in otherwise healthy individuals [1,2]. The most common causative organism is group A streptococcus (GAS) [3]. Most infections caused by GAS are common, superficial, and relatively mild such as impetigo and pharyngitis. However, these infections trigger immune sequelae such as rheumatic heart disease [4].

In some cases, GAS infections can be severe, causing STSS and necrotizing soft tissue infections. These cannot be treated effectively without prompt intensive care and debridement if necessary [5].

In Japan, appropriate notification measures based on the Infectious Disease Control law are mandatory for cases of STSS caused by β-hemolytic streptococcus. The number of infections is publicly available in statistical reports. STSS cases have increased since 2022 and doubled in 2024 as compared to the previous year.

Amid this growing outbreak, our institution encountered its first case of STSS in 2024. Here, we describe two STSS cases that followed a fall in an assisted living facility and cellulitis.

## Case presentation

Case 1

**Figure 1:**
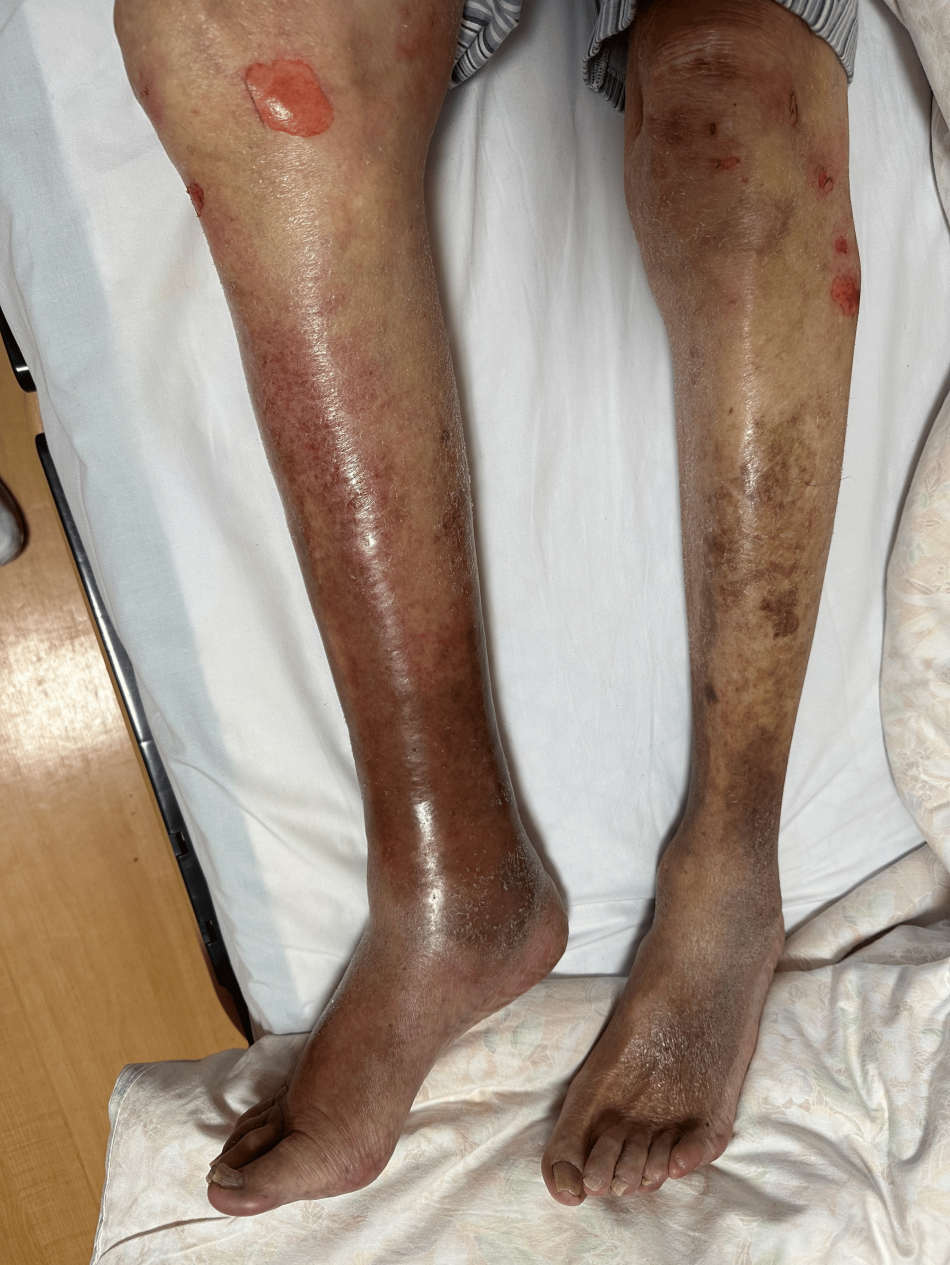
Lesion on the right lower limb of the first case (day of admission) Redness and swelling were observed in the right calf. No apparent necrosis was noted. Subsequently, vesicles formed and ruptured.

We present a case of a 93-year-old male with a medical history of angina pectoris, hypertension, benign prostate hyperplasia, and strangulating intestinal obstruction. He lived in an assisted living facility affiliated with our hospital and was independent in his daily activities. The patient was seen in the emergency department with a chief complaint of fever (39.0 ºC), chills, and shivering. He stated that he had fallen a few days ago. Physical examination revealed clear inflammatory signs in the right leg but no obvious evidence of trauma. He complained of only mild pain corresponding to the reddened area. He also had malaise. The findings did not suggest a necrotizing soft tissue infection (Figure 1). He did not have hypotension at that time.

Laboratory results on admission are illustrated in Table 1. Tests demonstrated a decreased leukocyte count, an elevated C-reactive protein (CRP) level, and a creatine phosphokinase (CPK) level. Liver and kidney dysfunction were also seen.

**Table 1:** Laboratory findings of the first case

Test	Values	Reference range and unit
White blood cell (WBC)	2560	3300-8600/μL
Haemoglobin (Hb)	15.5	13.7-16.8 g/dL
Platelets	105000	158000-348000/μL
Sodium (Na)	139	138-145 mmol/L
Potassium (K)	3.4	3.6-4.8 mmol/L
Chloride (Cl)	102	101-108 mmol/L
Blood urea nitrogen (BUN)	43.1	8-20 mg/dL
Creatinine	1.49	0.65-1.07 mg/dL
Estimated glomerular filtration rate (eGFR)	34	≥ 60.0
Total bilirubin	2.1	0.4-1.5 mg/dL
Aspartate aminotransferase (AST)	222	13-30 U/L
Alanine transaminase (ALT)	54	10-42 U/L
Alkaline phosphatase (ALP)	73	38-113 U/L
Lactate dehydrogenase (LDH)	473	124-222 U/L
γ-Glutamyl transpeptidase (γ-GTP)	8	13-64 U/L
Creatine kinase (CK)	10500	59-248 U/L
C-reactive protein (CRP)	19.50	0.00-0.14 mg/dL

The patient was hospitalized for cellulitis of the right lower limb with multiple organ dysfunction, and antibiotic therapy with cefazoline (1 g, intravenous, every 8 hours) and clindamycin (600 mg, intravenous, every 8 hours) was initiated. Infusion of crystalloid solutions was also performed to stabilize systemic hemodynamics and maintain sufficient urine output. However, he had hypotension, an altered state of consciousness, and respiratory failure 25 hours after admission. He was still febrile at 38.8 ºC and although the area of redness and swelling in the lower limb remained unchanged, we observed vesicle formation. Due to impaired consciousness, pain assessment was difficult. Considering the patient's age, we decided to limit treatment to supportive care. Approximately 30 hours after admission, the patient passed away. Blood culture results returned positive on the day following death, identifying GAS (Table 2). Based on these findings, a definitive diagnosis of STSS was made.

**Table 2:** Antimicrobial susceptibility test in the strain isolated from the first patient

Antimicrobial agent	MIC (μg/dL)
Penicillin G potassium	≤ 0.25
Ceftriaxone	≤ 0.06
Cefdinir	≤ 0.5
Cefcapene	≤ 0.25
Cefditoren pivoxil	≤ 0.25
Cefozopran	≤ 0.25
Imipenem / Cilastatin	≤ 0.12
Meropenem	≤ 0.25
Levofloxacin	2
Garenoxacin	1
Clarithromycin	> 0.5
Vancomycin	≤ 1

Case 2

**Figure 2:**
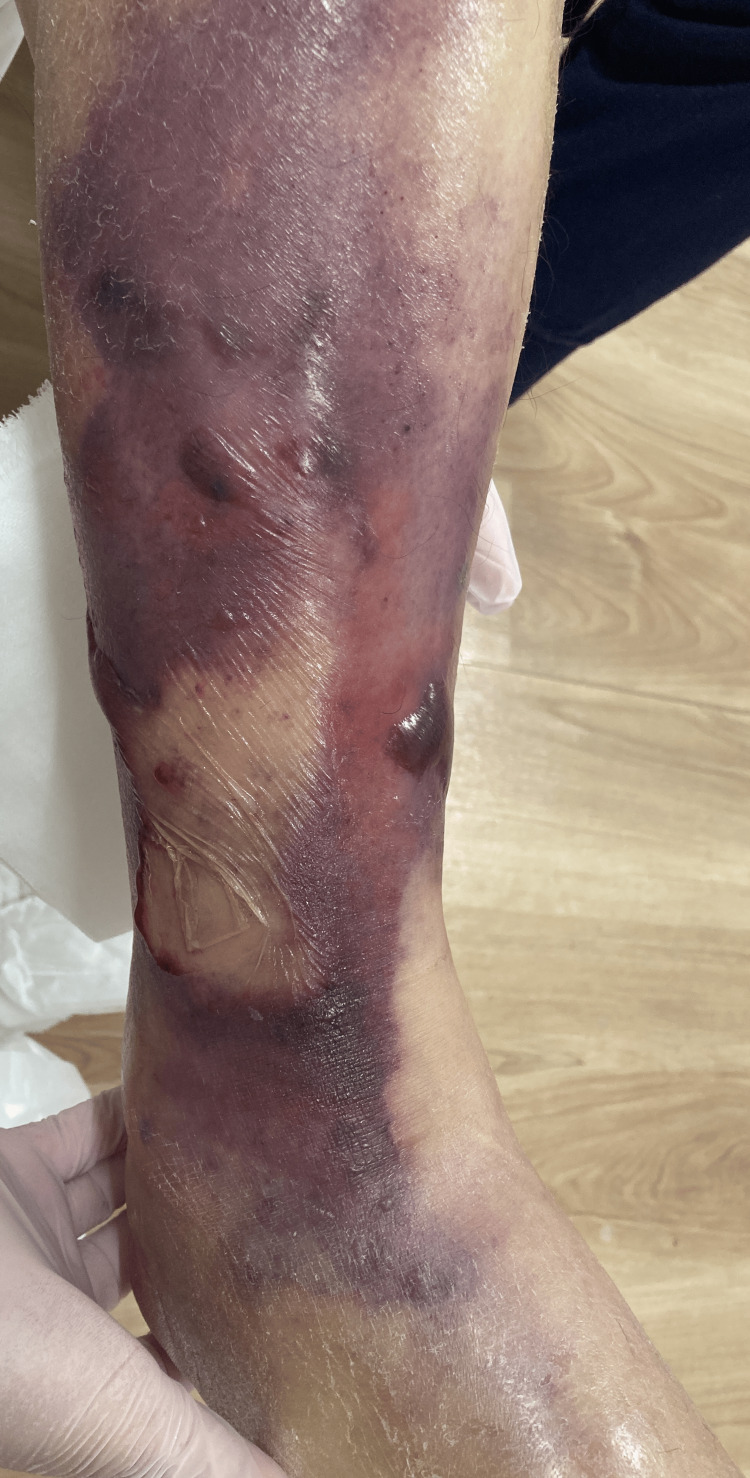
Lesion on the right lower limb of the second case (day of admission) Redness and swelling worsened compared to the previous day, with some areas forming vesicles. Some regions appeared purplish, suggesting necrosis.

The second case is a 72-year-old male with a history of type 2 diabetes mellitus, angina pectoris, Alzheimer's disease, and bipolar affective disorder. He lived in the same assisted living facility. One day before his hospital visit, he fell in his room and hit his right leg. The next day, he developed a fever (38.5 ºC) and a swollen right leg. Oral cefaclor (250 mg, every 8 hours) was initiated with the diagnosis of cellulitis. At the next follow-up visit on the following day, we observed findings that suggested cellulitis progression and suspected skin and soft tissue necrosis (Figure 2). He complained of severe pain in his lower limb. Laboratory tests revealed a decreased leukocyte count and an elevated CRP level (Table 3). The patient was referred to a critical care medical center. A rapid test conducted at the center was positive for Streptococcus pyogenes, and amputation and debridement were performed immediately. Antibiotic therapy with ampicillin (1 g, intravenous, every 12 hours) and clindamycin (600 mg, intravenous, every 8 hours) was also initiated. He recovered after two days of treatment and was transferred from the intensive care unit. He temporarily recovered and was discharged but later developed hospital-acquired pneumonia and passed away on postoperative day 18. The culture of the necrotic tissue revealed GAS (Table 4).

**Table 3:** Laboratory findings of the second case

Test	Values	Reference range and unit
White blood cell (WBC)	3010	3300-8600 /μL
Haemoglobin (Hb)	10.8	13.7-16.8 g/dL
Platelets	165000	158000-348000 /μL
Sodium (Na)	139	138-145 mmol/L
Potassium (K)	3.7	3.6-4.8 mmol/L
Chloride (Cl)	103	101-108 mmol/L
Blood urea nitrogen (BUN)	27.8	8-20 mg/dL
Creatinine	0.56	0.65-1.07 mg/dL
Estimated glomerular filtration rate (eGFR)	107	≥ 60.0
Total bilirubin	1.4	0.4-1.5 mg/dL
Aspartate aminotransferase (AST)	39	13-30 U/L
Alanine transaminase (ALT)	32	10-42 U/L
Alkaline phosphatase (ALP)	97	38-113 U/L
Lactate Dehydrogenase (LDH)	226	124-222 U/L
γ-Glutamyl Transpeptidase (γ-GTP)	44	13-64 U/L
Creatine kinase (CK)	429	59-248 U/L
C-reactive protein (CRP)	22.10	0.00-0.14 mg/dL

**Table 4:** Antimicrobial susceptibility test in the strain isolated from the second patient

Antimicrobial agent	MIC (μg/mL)
Ampicillin	≤ 0.25
Sulbactam / Ampicillin	≤ 0.25
Tazobactam / Piperacillin	≤ 1
Cefazolin	≤ 0.5
Ceftriaxone	≤ 0.5
Cefozopran	≤ 0.5
Amikacin	> 32
Arbekacin	> 8
Levofloxacin	2
Minocycline	≤ 1
Meropenem	≤ 0.12
Clarithromycin	> 8
Clindamycin	≤ 0.25
Linezolid	1
Vancomycin	≤ 0.5
Teicoplanin	≤ 1

## Discussion

STSS is a severe invasive infection characterized by the sudden onset of shock, multiple organ failure, rapid progression, and high mortality [3]. GAS is widely recognized as a pathogen that induces inflammatory diseases in humans, causing localized infections, such as pharyngitis and tonsillitis, as well as systemic infections, including scarlet fever and puerperal fever. In some cases, post-infectious immune responses produce anti-GAS antibodies that cross-react with human tissue components, potentially resulting in severe complications such as acute glomerulonephritis and rheumatic fever. However, due to the high efficacy of penicillin-based antibiotics, GAS infections have generally received limited attention. STSS was first reported in the United States in 1987, with the first documented typical case in Japan reported in 1992. Commonly referred to as "flesh-eating disease," this condition has recently garnered significant attention due to its severe clinical manifestations and high mortality rate [6].

The underlying mechanism of GAS is still unclear. Still, the virulence factors of GAS are remarkably diverse compared to those of other bacteria, and even among GAS strains, the specific virulence factors they harbor vary. It has been suggested that the absence of polymorphonuclear leukocytes (PMNs) at the site of infection plays a crucial role in the pathogenesis of severe invasive GAS infections. Streptolysin O-induced necrosis of PMNs and the cleavage of interleukin-8 (IL-8) by serine protease ScpC, which inhibits PMN migration, have been implicated as potential mechanisms contributing to disease severity [7]. A characteristic pathological feature of STSS is shock, which is thought to be triggered, in part, by superantigens. GAS produces multiple types of superantigens, which non-specifically activate a large number of T cells, leading to the excessive release of cytokines and subsequent development of shock [8].

Persons at increased risk for STSS include persons with underlying diabetes, cancer, human immunodeficiency virus infection, chronic lung or heart disease, and immunocompromising conditions [9,10]. However, it can also develop in healthy individuals [11], and the full pathogenesis remains unclear. In our cases, both cases had a history of heart disease, and the second case also had a history of diabetes, which is consistent with previous reports.

In our cases, both cases had cellulitis in the leg after falls. Both patients had clear histories of falls, but no apparent lower limb trauma. However, it is believed that the pathogen entered through a micro-wound sustained during a fall, leading to cellulitis, which subsequently triggered STSS, due to immunological vulnerability associated with advanced age, diabetes, and cardiovascular disease. In the first case, the patient had liver dysfunction, renal dysfunction, and rhabdomyolysis. This indicates that superantigens and inflammatory cytokines caused damage to the endothelial cells, leading to multiorgan dysfunction and resulting in a life-threatening condition. In the second case, the worsening of local findings in the lower limb was more significant than the deterioration of the patient's laboratory data. This may be the result of rapid pathogen proliferation due to the absence of PMNs at the local site. STSS progresses to severe disease through multiple mechanisms, and its clinical manifestations vary among patients.

STSS should be considered in the differential diagnosis even if the severe patients do not have obvious wounds [12]. Despite initially exhibiting only mild cellulitis-like findings, the first case was diagnosed with STSS. The absence of severe inflammatory findings may reflect the previously described characteristic of GAS, which inhibits PMN migration. STSS should be considered regardless of the severity of skin findings. There have been no reports of STSS caused by trauma from falls before. This report emphasizes that special attention is required in facilities like ours, which include an assisted living facility for elderly individuals.

**Figure 3:**
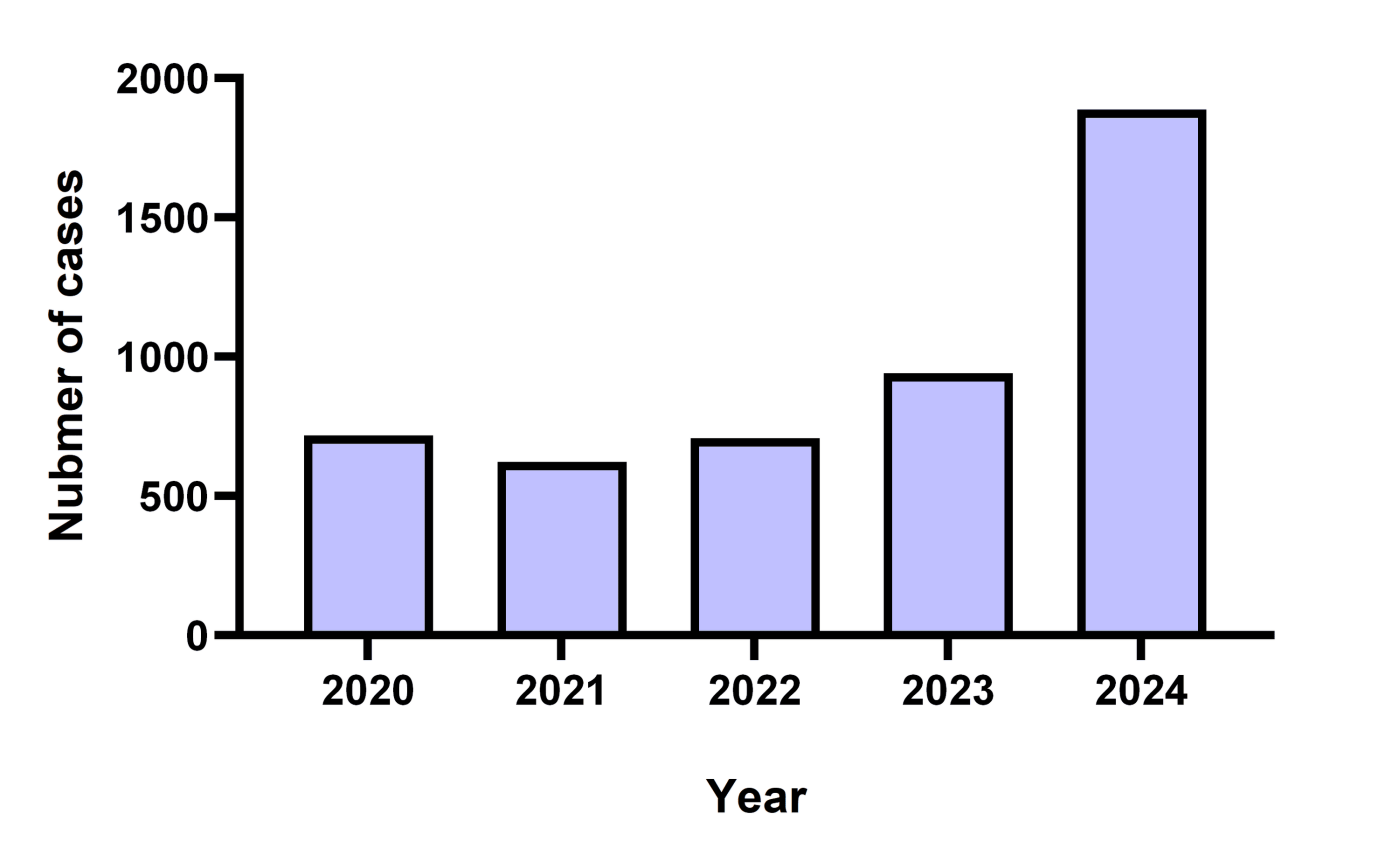
The number of streptococcal toxic shock syndrome (STSS) cases reported by the Ministry of Health, Labour, and Welfare of Japan from 2020 to 2024 Based on the Infectious Disease Control law in Japan, physicians are obligated to notify all cases of STSS caused by β-hemolytic streptococcus. Surveillance data is available on the website of the National Institute of Infectious Diseases (NIID). The authors created this chart based on data publicly available on the NIID website (https://www.niid.go.jp/niid/en/data.html)

Japan is experiencing a sharp increase in STSS cases, surpassing the previous year's statistics. According to Japan's Ministry of Health, Labour, and Welfare, there were 1,888 STSS cases in 2024, compared to 941 in 2023 (Figure 3) [13]. This surge is believed to be linked not only to the relaxation of COVID-19 precautions but also to the prolonged period of reduced social interactions and limited exposure to the natural environment [14].

Considering the current spread of STSS in Japan, we can encounter STSS in any medical institution. Early detection and intervention, including intensive care and surgery for necessary cases, are essential for prompt consultation and appropriate treatment.

## Conclusions

STSS is considered a rare but serious illness with variable mortality, and its exact mechanism remains unclear. We encountered two cases of STSS followed by cellulitis after a fall. Both cases followed a fatal course, but their clinical characteristics differed. The highlights of these cases are that both STSS cases occurred after their clear history of falls and cellulitis. Given the current spread of STSS in Japan, we may come across it in any medical institution. Early detection and intervention are crucial for prompt medical consultation and appropriate treatment. Since STSS can arise following trauma and minor injuries, caregivers in assisted living facilities for elderly individuals, who are at risk for falls, need to have special attention.
